# Oxidative stress induced by UVA photoactivation of the tryptophan UVB photoproduct 6-formylindolo[3,2-*b*]carbazole (FICZ) inhibits nucleotide excision repair in human cells

**DOI:** 10.1038/s41598-017-04614-8

**Published:** 2017-06-27

**Authors:** Reto Brem, Peter Macpherson, Melisa Guven, Peter Karran

**Affiliations:** The Francis Crick Institute, Midland Road, London, NW1 1AT UK

## Abstract

Potentially mutagenic DNA lesions induced by UVB (wavelengths 280–320 nm) are important risk factors for solar ultraviolet (UV) radiation-induced skin cancer. The carcinogenicity of the more abundant UVA (320–400 nm) is less well understood but is generally regarded to reflect its interaction with cellular chromophores that act as photosensitisers. The arylhydrocarbon receptor agonist 6-formylindolo[3,2-*b*] carbazole (FICZ), is a UVB photoproduct of tryptophan and a powerful UVA chromophore. Combined with UVA, FICZ generates reactive oxygen species (ROS) and induces oxidative DNA damage. Here we demonstrate that ROS generated by FICZ/UVA combinations also cause extensive protein damage in HaCaT human keratinocytes. We show that FICZ/UVA-induced oxidation significantly inhibits the removal of potentially mutagenic UVB-induced DNA photolesions by nucleotide excision repair (NER). DNA repair inhibition is due to FICZ/UVA-induced oxidation damage to the NER proteome and DNA excision repair is impaired in extracts prepared from FICZ/UVA-treated cells. NER protects against skin cancer. As a likely UVB photoproduct of intracellular tryptophan, FICZ represents a *de facto* endogenous UVA photosensitiser in sun-exposed skin. FICZ formation may increase the risk of solar UV-induced skin cancer by promoting photochemical damage to the NER proteome and thereby preventing the removal of UVB-induced DNA lesions.

## Introduction

Solar ultraviolet (UV) radiation causes skin cancer. UVB photons (wavelength 280–320 nm) are absorbed by DNA and introduce potentially mutagenic lesions in the form of cyclobutane pyrimidine dimers (CPDs) and pyrimidine (6-4)-pyrimidone photoproducts (6-4PPs). UVB exposure is firmly linked to sunlight-related cancer and the transitions at dipyrimidine sites that are regarded as the signature mutations of UVB-induced DNA damage, are predominant in skin cancers^1^. The nucleotide excision repair (NER) system that removes UVB-induced DNA photolesions provides significant protection against mutation and cancer induction in sun-exposed skin. Inefficient NER in the genetic disorder xeroderma pigmentosum (XP) is associated with a greatly increased susceptibility to sun-induced skin damage, mutation and skin cancer (reviewed in ref. 2). UVA (320–400 nm) is about 20-times more abundant than UVB and comprises over 95% of incident UV radiation. It is, however, poorly absorbed by DNA and most UVA-induced damage to DNA and other skin biomolecules is caused indirectly *via* interaction with intracellular photosensitisers that trigger the generation of reactive oxygen species (ROS)^3^. These endogenous UVA chromophores have not been fully characterised although porphyrins, flavins and melanin are among the potential candidates.

Tryptophan is an essential amino acid and is present in human serum at around 50–100 µM^4, 5^. It is photoactive and has a broad absorbance maximum at around 280 nm. Free tryptophan therefore represents a significant intracellular chromophore for the solar UV wavelengths that are incident on human skin. 6-formylindolo[3,2-*b*]carbazole (FICZ) is a major UVB photoproduct of tryptophan both *in vitro* and in irradiated human cells^6^. It is a potent agonist of the arylhydrocarbon receptor (AhR), a transcriptional activator that upregulates a number of stress-related genes^7^ including members of the MAPK signalling cascade^8^. The UVB-induced expression of AhR targets in human skin identifies FICZ as a likely photoproduct in sun-exposed skin cells.

Independently of its role in AhR activation, FICZ itself is photoactive. It has significant absorbance of both UVA and visible (blue) wavelengths. In cultured HaCaT keratinocytes, FICZ and UVA act synergistically to induce the expression of genes associated with oxidative and proteotoxic stress and to impair mitochondrial transmembrane potential. Consistent with the generation of oxidative stress, the combination of FICZ and UVA triggers the production of ROS (including singlet oxygen, ^1^O_2_) and consequently induces the formation DNA 8-oxo-7,8-dihydroguanine (8-oxoG)^9^. This potentially mutagenic DNA lesion can be excised by the base excision repair (BER) system initiated by the hOGG-1 DNA glycosylase.

Like FICZ, photosensitising drugs can interact with UVA to generate ROS and DNA 8-oxoG. Among the acknowledged pharmaceutical photosensitisers, the thiopurines 6-mercaptopurine and 6-thioguanine (6-TG)^10^ and the fluoroquinolone antibiotics ciprofloxacin and ofloxacin^11^ all generate ROS in UVA-dependent reactions. In addition to inducing DNA damage including 8-oxoG, UVA photoactivation of 6-TG and the fluoroquinolones also causes widespread damage to proteins^12, 13^. Of particular significance in the context of the skin cancer risk entailed by solar UV exposure, DNA repair proteins including PCNA and RPA, are among those inactivated by oxidation and these photosensitiser/UVA combinations inhibit BER and NER^12–14^.

In the case of intracellular tryptophan, the UV wavelengths in incident solar radiation can therefore both produce (from UVB) and activate (by UVA) FICZ to cause oxidative damage in skin cells. In order to determine whether UVA photoactivation of FICZ poses a threat to DNA repair and might thereby increase skin cancer risk, we have examined the effects of UVA/FICZ on NER and BER *in vivo* in cultured HaCaT keratinocytes and *in vitro* by biochemical assays.

## Results

### Protein oxidation and FICZ phototoxicity

**Figure 1:**
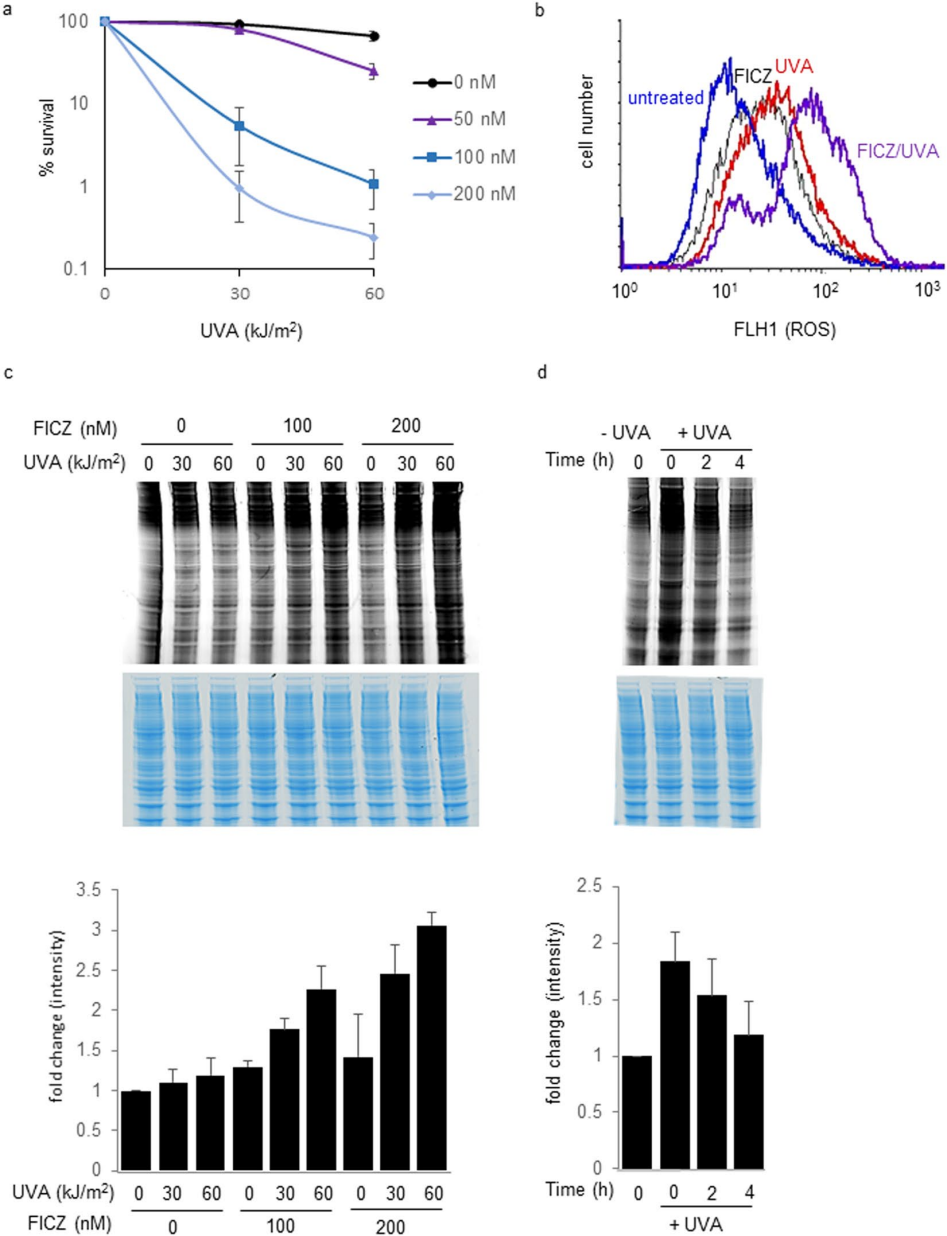
FICZ/UVA-induced cytotoxicity is associated with ROS generation and protein carbonylation. (**a**) Toxicity. HaCaT cells were treated with indicated doses of FICZ for 2 h and then irradiated with UVA. Survival of triplicate samples was determined by colony formation assay 10 days later. The means of three independent experiments are shown. (**b**) ROS induction. Cells were treated with 200 nM FIZC and irradiated with 60 kJ/m^2^ UVA. ROS levels (log scale) were analysed by FACS using the CM-H_2_CDFDA probe. (**c**) Protein carbonyl induction: Dose response. Cell extracts were prepared immediately after treating cells with the indicated doses of FICZ and UVA. Proteins (25 µg) were derivatised with Hydroxylamine Alexa Fluor 488 and separated by PAGE. Protein carbonyls were visualised at 488 nm (upper panel) and quantified using ImageQuant TL software (GE Heathcare). The gel was then stained with InstantBlue to provide a loading control (middle panel). The lower panel presents the mean (±SD) carbonyl values from three independent experiments. (**d**) Protein carbonyl turnover. Cells were treated with 200 nm FICZ for 2 h followed by irradiation with 60 kJ/m^2^ UVA. Protein carbonylation (upper panel) was assayed at the indicated times after irradiation. The gel was then InstantBlue stained (middle panel). The lower panel presents the mean (±SD) carbonyl values from three independent experiments.

Clonal survival assays indicated that low, non-toxic doses of UVA radiation (30, 60 kJ/m^2^) caused extensive death in HaCaT keratinocytes that had been treated with FICZ at concentrations ≥50 nM. Neither FICZ nor UVA alone was detectably cytotoxic (Fig. 1a). A 2 h exposure to FICZ (200 nM) or irradiation with UVA (60 kJ/m^2^) both induced a modest increase in intracellular ROS (median fluorescence 33 and 24 arbitrary units, respectively) compared to untreated cells (median fluorescence 14 units) whereas the effect of combined FICZ/UVA treatment was greater than additive (median fluorescence 72 units) (Fig. 1b). These observations confirm the previously reported synergistic effects of FICZ and UVA on toxicity and ROS induction in HaCaT cells^9^.

FICZ/UVA treatment caused extensive protein oxidation in HaCaT cells. Levels of protein carbonylation, one of the hallmarks of protein oxidation, increased in a FICZ concentration- and UVA dose-dependent manner (Fig. 1c). Consistent with a dependence on photochemically-induced ROS, treatment with both FICZ and UVA was required to induce widespread protein oxidation that under the most extreme conditions (200 nM/60 kJ/m^2^) reached around three times the level in untreated cells. Treatment with FICZ or UVA alone induced only small (≤30%) increases in protein carbonyl levels. Increased protein carbonylation was apparent immediately after UVA irradiation of FICZ-treated cells and declined during the 4 h post irradiation (Fig. 1d).

### DNA repair in FICZ/UVA-treated cells

#### DNA oxidation damage

**Figure 2:**
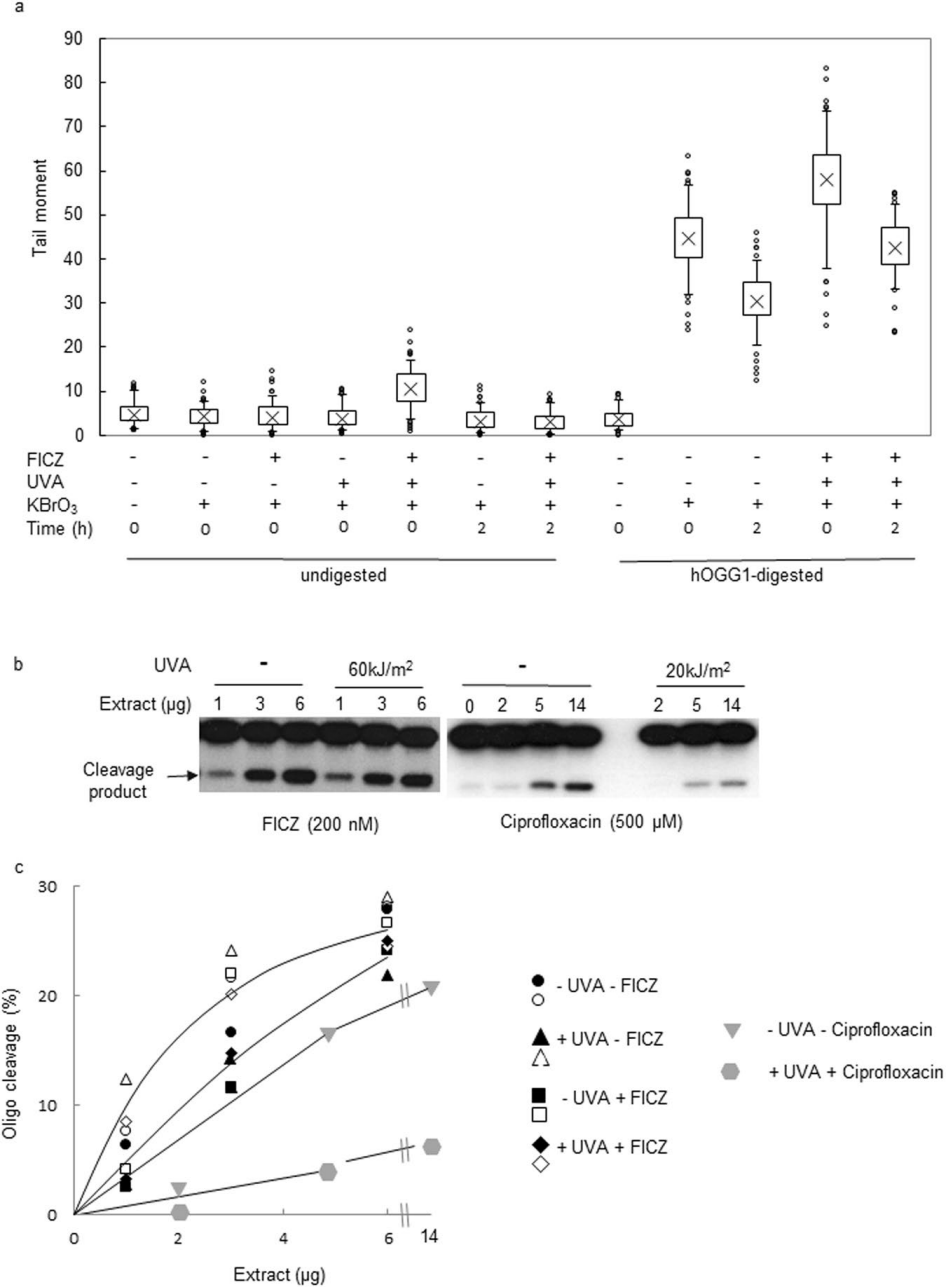
FICZ/UVA does not impair BER. (**a**) *In vivo* BER. HaCaT cells were treated with 200 nM FIZC (2 h) and 60 kJ/m^2^ UVA. After irradiation, they were exposed to 5 mM KBrO_3_ (20 min) and analysed by single cell electrophoresis directly after treatment or after re-incubation in normal medium for 2 hours to allow repair. To reveal DNA 8-oxoG, lysed cells were treated with recombinant human OGG-1 before electrophoresis. Comet tail moments of 100 cells per condition were measured and statistically analysed by the Comet IV software (Trevigen). (Cross = mean tail moment; Box = standard deviation; Bars = 90% of all analysed cells) (**b**) *In vitro* BER. Extracts prepared from HeLa cells that had been treated with FICZ (2 h) and UVA as shown were incubated (15 min, 37°) with an end-radiolabelled duplex oligonucleotide containing a single 8-oxoG:C basepair. Extracts were supplemented with purified *E.coli* endonuclease IV to ensure quantitative cleavage of apurinic sites generated by OGG-1. A similar assay of extracts prepared from HeLa cells that had been treated with ciprofloxacin/UVA is shown for comparison. OGG-1 generated cleavage products are indicated. Gels are cropped to display the only two radiolabelled oligonucleotide bands. Lower panels: Black and white symbols represent quantified data for cleavage by two independent sets of extracts from cells treated with FICZ/UVA as indicated. Grey symbols are values from the ciprofloxacin/UVA control. Since FICZ and/or UVA had no detectable effect on cleavage, a single line has been fitted by eye through the data from each set of assays.

DNA 8-oxoG is a major ROS-induced lesion that is repaired by BER initiated by the hOGG-1 DNA glycosylase. We have previously shown that some photosensitiser/UVA combinations decrease BER efficiency^12^. To examine whether FICZ/UVA treatment affects the efficiency of BER, we exposed FICZ/UVA treated HaCaT cells to potassium bromate (KBrO_3_) - an acknowledged source of DNA 8-oxoG^15^. DNA 8-oxoG levels were analysed by the alkaline comet assay with hOGG-1 digestion. Figure 2a shows that both KBrO_3_ and FICZ/UVA induce hOGG-1 sensitive sites in HaCaT DNA as revealed by a hOGG-1-dependent increase in tail moment. The high level of DNA 8-oxoG induced by a 20 min treatment with 5 mM KBrO_3_ was further modestly increased by treatment with FICZ/UVA confirming that FICZ/UVA also induces a low level of DNA 8-oxoG^9^. Consistent with active BER, DNA 8-oxoG levels declined during a post-treatment incubation of 2 h. During this time, the mean tail moment of cells treated either with KBrO_3_ alone or with KBrO_3_ plus FICZ/UVA decreased to similar extents. We conclude that BER of DNA 8-oxoG by HaCaT cells is not detectably affected by FICZ/UVA treatment.

The comet assay with hOGG-1 provides an indirect indication of cellular hOGG-1 DNA glycosylase activity. To investigate directly the effect of FICZ/UVA treatment on cellular hOGG-1, we assayed the hOGG-1 DNA glycosylase in extracts of FICZ/UVA-treated HeLa cells using an end-radiolabelled 23-mer duplex oligonucleotide containing a single 8-oxoG:C base pair^12^. Excision of the 8-oxoG generates an apurinic site that is quantitatively cleaved by a specific AP endonuclease to generate a labelled 13-mer that is resolved by denaturing PAGE. Figure 2b shows a typical assay and Fig. 2c presents the quantified data from assays performed with two independent sets of cell extracts. These *in vitro* assays revealed that hOGG-1 activity is not detectably affected by FICZ/UVA treatment. As a positive control for the effects of oxidation on BER, we examined hOGG-1 activity in extracts of cells treated with ciprofloxacin/UVA. This combination has previously been shown to damage the DNA repair proteome^13^. Contrary to FIZC/UVA treated cells, hOGG-1 activity was significantly diminished in extracts of ciprofloxacin/UVA-treated HeLa cells. The finding of unimpaired hOGG-1 activity in FICZ/UVA-treated extracts supports the data from the comet assay and provides confirmation that BER of DNA 8-oxoG is not detectably impaired by this treatment.

#### UV photoproducts

**Figure 3:**
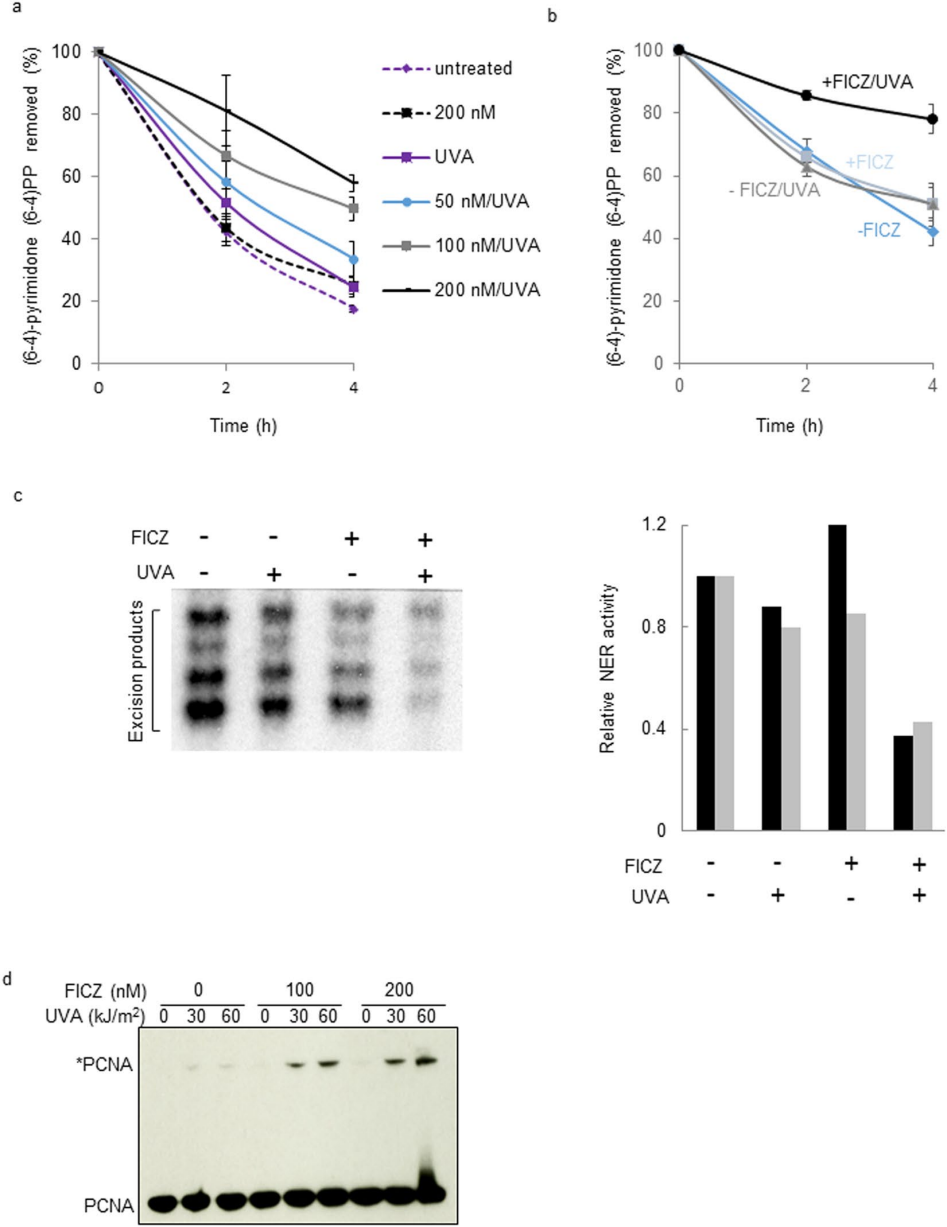
FIZC/UVA inhibits NER. (**a**) Removal of UVC induced photoproducts. HaCaT cells were treated with the indicated doses of FICZ for 2 h followed by 60 kJ/m^2^ UVA and 20 J/m^2^ UVC. DNA was extracted at times indicated and pyrimidine (6-4)-pyrimidone (6-4)PP photoproducts were measured by ELISA on triplicate samples. Data are the means of three independent experiments. Initial (Time = 0) ELISA values for all conditions agreed to ± 10% confirming that FICZ/UVA treatment does not induce measurable numbers of 6-4PPs. (**b**) Removal of UVB induced photoproducts. Cells were treated with 200 nM FICZ, 60 kJ/m^2^ UVA and 200 J/m^2^ UVB. Photoproduct excision was measured as in (**a**). (**c**) *In vitro* NER. Excision by NER of a single cisplatin adduct from a covalent circular duplex was analysed in extracts prepared from HeLa cells that had been treated with FICZ (1 h, 200 nM) and irradiated with UVA (60 kJ/m^2^). Excision products were end-radiolabelled and analysed by gel electrophoresis (left panel). Excision activity was quantified and data from two independent sets of extracts (represented by the black and grey bars) are presented in the right panel. (**d**) PCNA damage. HaCaT cells were treated with FICZ and UVA at the doses indicated. Protein extracts were prepared immediately after irradiation and analysed by western blotting. Crosslinked PCNA (PCNA*) is indicated.

Direct assays of photoproduct removal indicated that FICZ/UVA treatment does impair nucleotide excision repair (NER) by HaCaT cells. Figure 3a shows that treatment with combined FICZ and UVA (60 kJ/m^2^) induced a concentration-dependent inhibition of NER of the DNA 6-4PPs induced by 20 J/m^2^ UVC. At the highest FICZ concentration (200 nM), FICZ/UVA treated cells excised only approximately 40% of UVC-induced 6-4PPs in the 4 h after irradiation. In contrast, neither 200 nM FICZ nor 60 kJ/m^2^ UVA alone detectably affected NER efficiency and FICZ-treated, UVA-irradiated and unirradiated HaCaT cells all excised approximately 80% of UVC-induced DNA 6-4PPs within 4 h. FICZ/UVA treatment also inhibited NER of 6-4PPs induced by UVB. In untreated or UVA treated cells, around 40% of the UVB-induced lesions were removed in the 2 h after irradiation whereas FICZ/UVA treated cells excised only around 15% of these photoproducts within the same time (Fig. 3b).

#### Protein damage mediates DNA repair inhibition after FICZ/UVA treatment

Assays of NER using protein extracts from FICZ/UVA treated HeLa cells indicated that protein damage was responsible for repair inhibition. The removal of an NER substrate cisplatin adduct from plasmids^16^ was impaired in extracts prepared from HeLa cells that had been treated with FICZ/UVA (Fig. 3c). In the representative experiment shown in Fig. 3c (left panel), FICZ (200 nM)/UVA (60 kJ/m^2^) caused significant inhibition of lesion removal by NER. GelDoc quantification of data from two independent sets of cell extracts (Fig. 3c, right panel) confirmed that this FIZC/UVA treatment inhibited NER activity by approximately 60%.

FICZ/UVA damaged the PCNA DNA replication and repair complex (Fig. 3d). The PCNA homotrimer is particularly susceptible to oxidation and undergoes covalent crosslinking between the 33 kDa subunits even under relatively mild oxidising conditions^17^. The product of PCNA crosslinking, PCNA*, is just detectable after exposure of HaCaT cells to low (30, 60 kJ/m^2^) UVA doses. Treatment with FICZ enhanced UVA-mediated PCNA* formation in a concentration-dependent manner. FICZ alone did not induce detectable PCNA* formation. The PCNA species that migrates slightly more slowly than the PCNA monomer and is present after the most severe FICZ/UVA treatment may be monoubiquitinated PCNA that is not fully resolved from the unmodified PCNA monomer. The presence of this modified PCNA suggests that FICZ/UVA induces the replication blocking DNA lesions that are required to trigger PCNA monoubiquitination.

**Figure 4:**
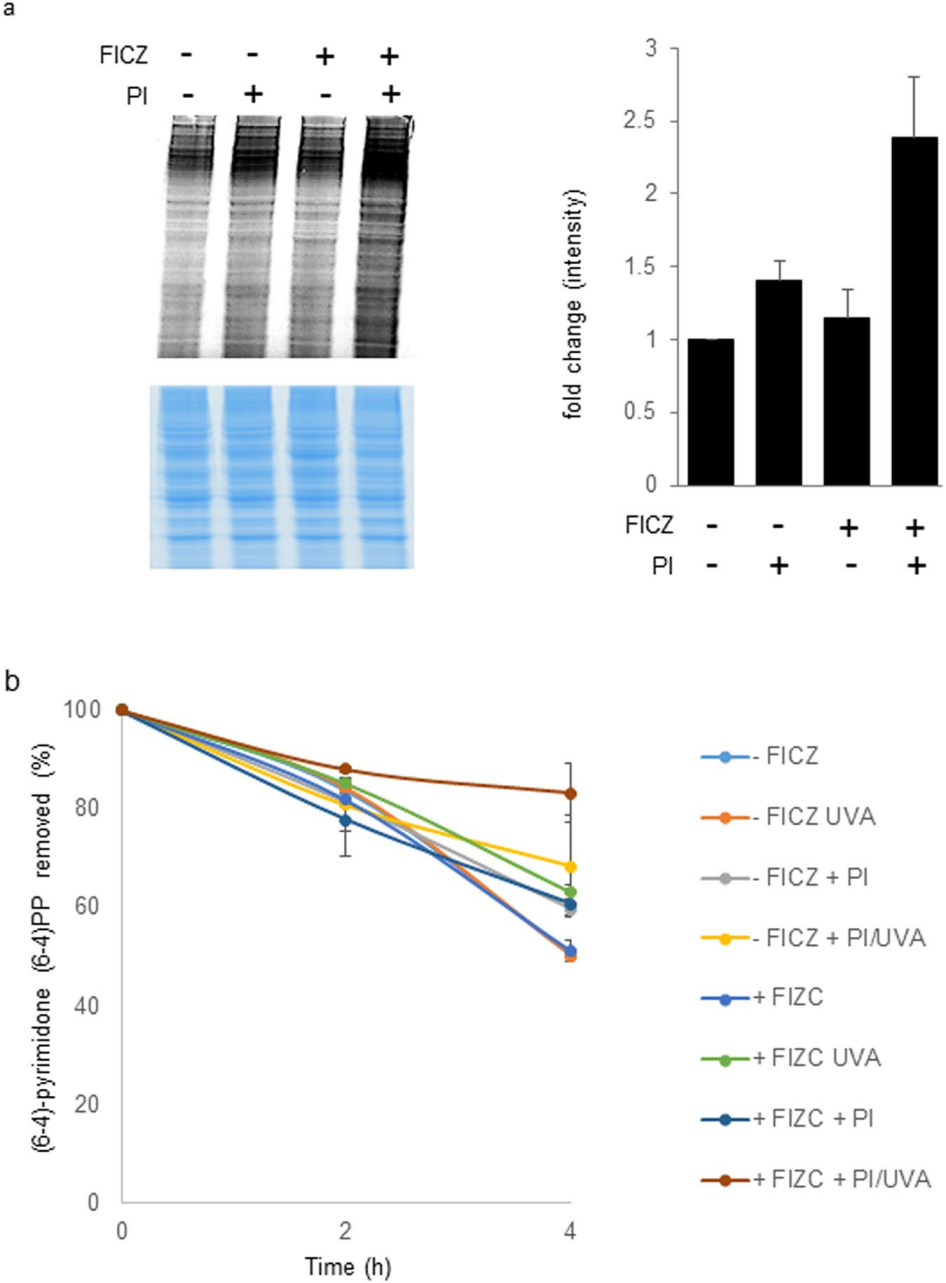
Proteasome inhibitors (PI) enhance protein carbonyl retention and NER inhibition by FICZ/UVA. (**a**) HaCaT cells were pre-treated with lactacystine and MG-132 (10 µM each) for 3 h in complete medium prior to the addition of 100 nM FICZ as indicated. After 2 h, cells were irradiated with UVA (30 kJ/m^2^) and returned to growth medium containing the proteasome inhibitors as indicated. After a further 4 h incubation, cell extracts were prepared and protein carbonyls were derivatised with Hydroxylamine Alexa Fluor 488 and analysed as in Fig. 1. The gel was InstantBlue stained to provide a loading control (lower panel). The right hand panel presents the mean (±SD) protein carbonyl values from three independent experiments. (**b**) *In vivo* NER. HaCaT cells pretreated with proteasome inhibitors and treated with FICZ as above were irradiated with UVA (30 kJ/m^2^), immediately followed by 200 J/m^2^ UVB. Irradiated cells were returned to normal growth medium containing the proteasome inhibitors and DNA was extracted at indicated times. 6-4PPs were measured by ELISA on triplicate samples. Data are the means of three independent experiments.

PCNA crosslinking is one example of a generalized protein oxidation induced by FICZ/UVA. The accumulation of oxidised proteins is generally detrimental to the well-being of cells. More specifically, oxidised enzymes are less efficient and oxidised proteins are prone to form aggregates that impair cellular processes (reviewed in ref. 18). To examine the effect of a persistently high level of oxidised proteins on NER efficiency, HaCaT cells were treated with proteasome inhibitors to prevent the turnover of oxidised proteins. Inhibition of the 20S proteasome by lactacystin and MG-132 prolonged the persistence of high levels of FICZ/UVA-induced protein carbonyls (Fig. 4a) and enhanced the inhibitory effect of FICZ/UVA treatment on NER (Fig. 4b). In the experiments depicted in Fig. 4b, HaCaT cells which had been treated with 100 nM FICZ and 30 kJ/m^2^ UVA removed around 35% of UVB-induced 6-4PPs in the 4 h following irradiation. This is a slight impairment (a decrease of 15%) compared to cells treated with UVB alone. Treatment with lactacystin and MG-132 for 3 h prior to and following UVB irradiation increased the impairment of NER and over 90% of the photoproducts remained in DNA 4 h after their induction.

## Discussion

Our findings demonstrate that the NER system that provides important protection against skin cancer is compromised in cells treated with UVA and the UVA/visible photosensitiser FICZ. NER inhibition reflects the widespread protein damage that is one consequence of the oxidative stress that photoactivated FICZ induces. Along with 6-TG^12, 14^ and the fluoroquinolone antibiotics^13, 14^, FICZ is therefore another example of a chromophore that interacts with UVA to induce widespread protein damage that compromises NER efficiency. We have previously reported that UVA-activated 6-TG and fluoroquinolones cause oxidation damage to replication and repair proteins. Complementation analysis of NER assays identified the RPA complex^12–14^ as a major target for damage leading to NER inhibition. In contrast to the exogenously supplied 6-TG or fluoroquinolones, FICZ can be considered a *de facto* endogenous UVA photosensitiser. It can be generated from tryptophan by increased oxidative stress conditions such as elevated H_2_O_2_ concentrations^19^ and thereby sensitise skin to solar UVA. The most important source of FICZ in skin is, however, likely to be photochemical as FICZ is a UVB photoproduct of tryptophan. In skin exposed to the full spectrum of incident solar radiation, FICZ is produced by UVB and responds to UVA and visible wavelengths to induce ROS that damage DNA and proteins. The extreme skin cancer proneness of xeroderma pigmentosum patients^2^ clearly implicates NER in the removal of potentially mutagenic and carcinogenic UVB-induced DNA photolesions. In NER-competent individuals, partial attenuation of NER mediated by FICZ photoactivation in chronically sun-exposed skin is a potential contributory factor to skin cancer risk.

The repair of DNA 8-oxoG, a major product of elevated oxidative stress, was not detectably impaired following FICZ/UVA treatments that induced significant NER inhibition. BER of 8-oxoG is vulnerable to oxidation under extreme conditions of oxidative stress and the hOGG-1 DNA glycosylase susceptible to inhibition by oxidation^20^. The reason for this more selective inhibition of DNA repair is currently unclear. It may reflect the relatively mild oxidative stress induced by the FICZ/UVA combinations we used and/or and potentially different types of ROS that this combination generates. An additional possible contributory factor is the relative target sizes of the NER and BER proteomes. NER involves the coordinated action of around 30 proteins^21^ whereas BER of DNA 8-oxoG is accomplished by less than one third of this number.

NER is a versatile DNA repair system and its inhibition by oxidation has implications beyond UV-induced DNA damage. In addition to its canonical UV photoproduct substrates, NER is active on a wide range of chemically-induced DNA lesions. This versatility underlies the protection that NER confers against the numerous chemical carcinogenesis that induce bulky DNA adducts. In addition to solar UV, smoking is also an acknowledged risk factor for skin cancer. Potentially mutagenic DNA adducts generated from polycyclic aromatic hydrocarbons such as benzo[a]pyrene (B[a]P), an important tobacco smoke carcinogen, are *bona fide* NER substrates^22^. In order to react with DNA, B[a]P requires metabolic activation by the cytochrome P450-1 family enzymes that are induced following AhR activation^23^. As a potent AhR agonist, FICZ stimulates B[a]P activation and synergistically enhances the formation of potentially mutagenic DNA adducts^24^. The UVB-mediated formation of FICZ therefore increases the likelihood that potentially mutagenic DNA lesions will be generated from the group of potent chemical carcinogens to which B[a]P belongs. If the efficiency of NER is simultaneously compromised by UVA photoactivation of FICZ, the two independently generated effects of increased DNA damage and decreased rate of removal will combine to increase skin cancer risk.

In summary, our study defines FICZ as the first *de facto* endogenous UVA/visible photosensitiser that causes inhibition of NER, the DNA repair pathway that provides the most important protection against skin cancer.

## Methods

### Cell culture, survival and ROS measurement

Cells were cultured in DMEM supplemented with 10% foetal calf serum. Treatment with 6-formylindolo(3,2-b)carbazole (FICZ, Sigma) was for 2 h prior to UVA irradiation. All cell lines had been authenticated by STR profiling within the twelve months prior to use. To assay cell survival, treated cells were seeded in triplicate into 6-well plates at a density of 300 cells/well and the survival determined by colony counting 10 days later. Survival experiments were repeated three times. To measure intracellular ROS, cells were incubated in normal medium containing CM-H_2_CDFDA (Life Technologies) for 20 min at 37 °C before irradiation and FACS analysis using a Becton Dickinson FACS Calibur and CELLQUEST software.

### UV irradiation

Cells were irradiated in PBS. UVA was delivered using a UVH 250 W iron bulb (UV Light Technology Limited, emission maximum 365 nm) at a dose rate of 0.1 kJ/m^2^/s. UVB radiation (maximum 312 nm) was from a LF-215 60 W bulb (Uvitec Limited) at 5 J/m^2^/s. 254 nm UVC was delivered by a Stratalinker UV Crosslinker (Stratagene) at 10 J/m^2^/s.

### Protein carbonyl detection

Cell extracts were prepared in RIPA buffer. Protein carbonyls were derivatised by incubating extracts (25 µg protein) with 50 μg/ml Alexa Fluor 647 or 488 Hydroxylamine (FHA; Invitrogen) for 2 h at 37 °C. Following separation on 10% Bis-Tris polyacrylamide gels (Invitrogen), carbonylated proteins were visualised at 633 or 488 nm using a Typhoon scanner (GE Heathcare) and quantified using the ImageQuant TL software (GE Heathcare). The same gels were then stained with InstantBlue (Expedeon). Each experiment was repeated three times.

### Photoproduct repair

Irradiated cells were returned to full medium at 37 °C and sampled at different times. DNA was extracted using the QIAamp DNA mini kit (Qiagen) and 6:4 Py:Pys measured by ELISA (Cosmo Bio). Assays were performed on triplicate samples and from three independent sets of treated cells. *In vitro* dual-incision NER assays were performed according to the modified method of Laine *et al.* ^16^.

### Immunoblotting

Proteins were separated on 10% Bis-Tris gels and transferred to Immobilon-P membranes (Millipore). PCNA antibodies (1:1000) were from Abcam. Antigen-antibody complexes were visualised by ECL detection agent (GE Healthcare).

### Comet Assay

Alkaline comet assays were performed as described^25^. Targets of the OGG1 DNA glycosylase were revealed by washing cell lysates three times with glycosylase reaction buffer and then incubating them with recombinant human OGG-1 (Trevigen) (2 units/50 µl buffer per slide for 1 h at 37 °C) before electrophoresis. Control slides were treated with buffer alone.
